# Acceptability of the Dapivirine Vaginal Ring and Daily Oral Pre-exposure Prophylaxis (PrEP) during Pregnancy in Malawi, South Africa, Uganda, and Zimbabwe

**DOI:** 10.1007/s10461-024-04421-z

**Published:** 2024-07-25

**Authors:** Alinda M. Young, Marie C.D. Stoner, Florence Mathebula, Rebone Mohuba, Alejandro Baez, Linly Seyama, Prisca Mutero, Juliane Etima, Zayithwa Fabiano, Lee Fairlie, Ashley J. Mayo, Jennifer E. Balkus, Mei Song, Katherine Bunge, Jeanna Piper, Ivan C. Balan, Ariane van der Straten, Elizabeth T. Montgomery

**Affiliations:** 1 Women’s Global Health Imperative at RTI International, 2150 Shattuck Avenue, Berkeley, CA 94104 USA; 2 Department of Maternal and Child Health, University of North Carolina at Chapel Hill, Chapel Hill, NC USA; 3 Wits RHI, Faculty of Health Sciences, University of the Witwatersrand, Johannesburg, South Africa; 4 Johns Hopkins Project – Kamuzu University of Health Science, Blantyre, Malawi; 5 University of Zimbabwe – Clinical Trials Research Centre, 15 Phillips Road, Belgravia, Harare Zimbabwe; 6 Makerere University – Johns Hopkins University Research Collaboration, Upper Mulago Hill Road Kampala, Kampala, Uganda; 7 FHI 360, 359 Blackwell St., Suite 200, Durham, NC USA; 8 Department of Epidemiology, University of Washington School of Public Health, Seattle, USA; 9 Vaccine and Infectious Disease Division, Fred Hutch Cancer Center, Seattle, USA; 10 Magee-Women’s Hospital of UPMC, 300 Halket Street, Pittsburgh, PA USA; 11 University of Pittsburgh, Pittsburgh, PA USA; 12 DAIDS/NIAID/NIH, Bethesda, MD USA; 13 Department of Behavioral Science and Social Medicine, Florida State University College of Medicine, Tallahassee, FL USA; 14 ASTRA consulting, 256 Stanford Avenue, Kensington, CA USA; 15 Center for AIDS Prevention Studies (CAPS) UCSF, San Franscico, CA USA

**Keywords:** HIV/AIDS, Acceptability, Pregnant Persons, Oral PrEP, Dapivirine Vaginal Ring, Eastern and Southern Africa

## Abstract

Pregnant and lactating persons in sub-Saharan Africa face a heightened risk of HIV acquisition, due to biological and behavioral factors, combined with limited access to prevention and treatment services. Oral pre-exposure prophylaxis (PrEP) and the dapivirine vaginal ring are promising tools for HIV prevention, and the ring’s recent approval in multiple African countries represents a significant advancement in expanding access to HIV prevention. In a nested qualitative study within the MTN-042 trial, we explored the acceptability of study products among pregnant persons in the second and early third trimesters. Interviews were conducted privately, using a semi-structured guide with 77 participants, in participants’ preferred language. Topics explored included product acceptability (using the theoretical framework of acceptability), user experience, satisfaction, disclosure, community attitudes, and sexual activity during pregnancy. Interview transcripts were analyzed using Dedoose software. We observed positive attitudes among participants towards the study products, which they found generally user-friendly, despite the added complexities of using them during pregnancy. Participants recognized that consistent and correct use would provide protection for both them and their unborn children. Although initial concerns existed, most of these worries dissipated over time, with study staff support and increased product use experience. These findings emphasize the importance of continued surveillance, support, and education to ensure the successful rollout of new HIV prevention measures during pregnancy.

## Introduction

Pregnancy and lactation are periods that significantly increase risk of HIV acquisition in sub-Saharan Africa (SSA), particularly since those periods [1–4] might last many years. The combination of biological and behavioral factors, including the likelihood of sexually transmitted infections and limited access to HIV prevention and treatment services, contributes to increased risk during these times [1, 5–7]. Despite increased antiretroviral therapy (ART) coverage during pregnancy from 33% in 2010 to 69% in 2019, and a concurrent reduction in vertical transmission from 27 to 17% [8], there remains an ongoing need for more effective prevention and treatment strategies to eliminate maternal infection and subsequent vertical HIV transmission. Specifically, concerns remain that in some SSA regions, programs only reached about 53% of pregnant or lactating persons living with HIV in 2022 [9]. By developing targeted interventions meeting their specific needs and circumstances, it may be possible to further reduce the burden of HIV in SSA and improve health outcomes for pregnant persons and their children.

Oral pre-exposure prophylaxis (PrEP) is an effective tool for HIV prevention [3, 10, 11], and recent data have supported safety among pregnant and lactating persons [12, 13]. The World Health Organization (WHO) guidelines recommend that oral PrEP be offered to HIV uninfected pregnant persons to prevent vertical transmission [12–14]. The dapivirine vaginal ring (the ring) is used continuously for a month and has demonstrated safety and effectiveness against HIV when used consistently in non-pregnant persons [15, 16]. The ring received EMA positive opinion in July 2020 and was recommended by the WHO in their 2021 guidelines for high-risk persons [13, 14]. The ring is now approved for use in multiple African countries with ongoing regulatory approvals in others [17]. This represents an important step towards expanding access to effective HIV prevention options in SSA, particularly those who may face barriers to accessing or using daily oral PrEP [18, 19].

The MTN-042/DELIVER trial is a critical clinical trial assessing the safety, pharmacokinetics, adherence, and acceptability of the ring and oral PrEP among pregnant persons living without HIV in four African countries. As part of DELIVER, we sought to explore the acceptability of study products during pregnancy, particularly the ring, and explore changes in perceptions and usage patterns over time. We specifically investigated the last two cohorts in DELIVER, involving pregnant persons in the early and mid-stages of pregnancy, and assessed the impact of parity on their attitudes about the study products.

## Methods

### Clinical Trial Design and Study Population

MTN-042/DELIVER study, a two-arm, randomized, open-label Phase 3b trial in Blantyre, Malawi; Johannesburg, South Africa; Kampala, Uganda; and Chitungwiza, Zimbabwe, enrolled participants from February 2020 to January 2023. The study was designed to enroll pregnant persons in three cohorts defined by gestation period, starting with those at most advanced gestation, moving to earlier gestation with each cohort, with product use stopping at the time of their pregnancy outcome or 41 weeks of gestation [20]. Safety reviews were scheduled between each cohort before progression of enrollment into earlier pregnancy stages. Participants were eligible if they were living without HIV, between the ages of 18 and 40, and had an uncomplicated singleton pregnancy – further description of the study design are published elsewhere [20]. Cohort 1 (late third trimester) enrolled 150 participants between 36 and 37 weeks, cohort 2 (early third trimester) enrolled 157 participants between 30 and 35 weeks. Participants in both the late-and-early third trimester were randomized 2:1 to the ring and oral PrEP. Cohort 3 (second trimester) enrolled 251 participants between 12 and 29 weeks and randomized in a 4:1 ratio. More details of the trial design and population have been published elsewhere [20].

### Qualitative Study Design and Subsample

In-depth interviews (IDIs) were conducted with participants in each cohort to characterize acceptability of the ring and oral PrEP. Findings from late third trimester, which included up to 7 weeks of use, are reported elsewhere [21]. Within the remaining cohorts, participants were purposively recruited by site, product arm assignment and parity, for a total of 35 in early third trimester and 42 in second trimester. Parity was accounted for by recruiting a 1:1 ratio of persons with previous pregnancy healthy outcomes (referred to as parous) and those who were nulliparous or had experienced a pregnancy loss prior to 20 weeks (referred to as nulliparous). In each parity subgroup, persons were subsequently selected in a ratio to reflect cohort-associated arm assignment ratios (ring: oral PrEP). A few participants delivered before their IDI was conducted and were interviewed postpartum (2 in early third trimester and 3 in second trimester). Additionally, five participants were chosen for “special cases” IDIs, 3 from early third trimester and 2 from second trimester. This selection aimed to examine if product use differed in pregnant persons with depressive symptoms, which was explored further in another manuscript within the study [22].

IDIs were conducted at a single time for each participant by trained female interviewers at each clinic site using a semi-structured interview guide. Interviews were conducted face-to-face in private locations in the language of the participants’ choice (Chichewa, Luganda, Sesotho, isiZulu, Shona and/or English) and lasted an average of 69 min (range 30–140 min). Topics discussed included study product acceptability and user experience; overall satisfaction with assigned study product; disclosure and community attitudes; and sexual activity during pregnancy (see Table 1). In second trimester, we used a timeline tool to facilitate discussions about pregnancy experiences from conception knowledge up to the time of the IDI, to track items such as the first pregnancy awareness, symptoms, relationship changes, study/product usage initiation, and pregnancy progression. Signed informed consent forms approved by the relevant ethics committees were obtained prior to each IDI. All IDIs were audio recorded, transcribed verbatim, and translated into English by study site staff or through translation/transcription agencies and transcripts went through quality control process by study site interviewers and qualitative analysts before coding and analysis.

**Table 1:** IDI main topics and discussion Points*

Main IDI Guide Topics	Discussion Points
Experience with pregnancy and study participation	• Pregnancy journey • Use of HIV prevention methods other than study products during pregnancy • COVID-19 and effect on pregnancy • Motivation to join DELIVER • Initial feelings about study products • Pre-use feelings about assignment product
Product Acceptability, Attitudes, and Use Experience	• Initial experience with product use (e.g., first insertion/removal) • Current thoughts on the assigned study product • Challenges with product use • Facilitators to using assigned product consistently • Plans for removing ring before delivery • Experience with ring removal during delivery
Concerns about health and care-seeking during pregnancy	• Effect of product use on maternal health now and in the future • Effect of product use on baby’s health now and in the future • Delivery preparations (e.g. traditional birth preparations)
Disclosure and Community Views	• Disclosure to male partners • Disclosure to other people, besides male partners • Community views on the use of HIV prevention methods during pregnancy
Sexual activity during pregnancy	• Beliefs about sexual activity during pregnancy • Experiences with sexual activity in various pregnancy stages • Effect of product use on own or partner’s sexual desire/pleasure
Satisfaction with assigned product	• Satisfaction with study product in preventing HIV during pregnancy (e.g., likes and dislikes) • Desire to use study product in the future

*This table includes the overall discussion points in the IDI guide which will also inform proceeding analysis and not solely the points that were discussed in this manuscript

### Qualitative Analysis

Interview transcripts were uploaded into Dedoose software (version 9.0.17–9.0.90) and coded by a team of three U.S.-based qualitative analysts. Coding followed an iteratively developed codebook that designated codes corresponding to the research objectives and IDI guides, the study population, previous analyses [21], and literature regarding biomedical HIV prevention in these settings. The codebook included descriptive codes that directly corresponded to topical areas relevant to the study (e.g. product acceptability, side effects, preference, product attributes, effect on life, maternal health, fetus/baby health, disclosure, pregnancy experience). Over approximately eight months, bi-weekly coding meetings were conducted to assess intercoder reliability, reach consensus on code interpretation, and refine the codebook, as necessary.

After coding, key data excerpts related to product acceptability were summarized by a team of 6 U.S and South African-based analysts using a matrix-based approach to review the seven constructs (Affective Attitude, Burden, Ethicality, Intervention Coherence, Opportunity Costs, Perceived Effectiveness, and Self-Efficacy) of the Theoretical Framework of Acceptability (TFA) [23]. The TFA was used to investigate how these constructs influenced participants’ perspectives on product use. Matrix-based TFA data were additionally examined by site and parity to identify any salient differences. The narrative data from the timeline tool discussions were transcribed and included in the coding process described above. Notably, the results reflect more acceptability data for the ring compared to oral PrEP because of the 4:1 randomization scheme in the second trimester. The study adhered to the COREQ guidelines to ensure a rigorous qualitative methodology and transparency in reporting of findings [24].

### Positionality and Reflexivity Statement

To ensure quality and accuracy of data interpretation, the author list includes at least one author from each country site. As part of our reflexive practice, the IDI guides were reviewed by qualitative researchers at each of the study sites to ensure cultural relevance and appropriateness. The data was collected by local research scientists who have a deep understanding of the local cultural and political contexts. Furthermore, the study team has decades of experience conducting research in these settings. Lastly, the sites’ qualitative researchers, who also have decades of experience, received study-specific training on procedures and practiced administering the guides through role playing, to ensure a clear understanding in both English and the local languages.

## Results

The qualitative sub-study of DELIVER included a sample of 77 participants (35/157 for early third trimester and 42/251 for second trimester, see Table 2). The demographic characteristics of the qualitative participants sometimes varied across cohorts and overall study participants. The median age of the qualitative sample was 24, and this age was consistent across the two cohorts. Furthermore, by design, more than 75% of the sample belonged to the ring group. Most participants (94%) had a male partner who was the father of the baby, was aware of the participant’s enrollment in the study and knew that she would be using an HIV prevention product.

**Table 2:** Demographic characteristics of DELIVER second trimester and early third trimester overall and for the qualitative subsample

	MTN-042 early third trimester		MTN-042 second trimester	
	All (*N* = 157)	Qualitative Sample (*N* = 35)	All (*N* = 251)	Qualitative Sample (*N* = 42)
Age, median (interquartile range (IQR))	26 (22, 30)	24 (21, 29)	24 (22,29)	24 (21, 28)
Product Assignment, n(%)				
Oral PrEP	51 (32.5)	8 (22.9)	49 (19.5)	8 (19.0)
Vaginal Ring	106 (67.5)	27 (77.1)	202 (80.5)	34 (81.0)
Site, *n* (%)				
Blantyre, Malawi	40 (25.5)	9 (25.7)	66 (26.3)	10 (23.8)
Johannesburg, South Africa	28 (17.8)	10 (28.6)	44 (17.5)	11 (26.2)
Chitungwiza, Zimbabwe	47 (29.9)	8 (22.9)	73 (29.1)	11 (26.2)
Kampala, Uganda	42 (26.8)	8 (22.9)	68 (27.1)	10 (23.8)
Has a primary partner	153 (97.4)	35 (100)	242 (96.4)	37 (88.1)
Partner knows you are enrolled in a study	141 (92.2)	29 (82.9)	224 (92.6)	32 (86.5)
Partner knows you will be using an HIV prevention product, *n* (%)*	134 (87.6)	29 (82.9)	196 (81.0)	27 (73.0)
Partner is the father of the baby	149 (97.4)	35 (100)	239 (99.2)	36 (97.3)
Primary partner has other partners, *n* (%)*				
Yes, knows	21 (13.7)	4 (11.4)	26 (10.7)	3 (8.1)
Yes, suspects	16 (10.5)	3 (8.6)	39 (16.1)	8 (21.6)
No	40 (26.1)	10 (28.6)	59 (24.4)	7 (18.9)
Don’t know	76 (49.7)	18 (51.4)	118 (48.8)	19 (51.4)

*Among those who have a partner

### Acceptability Overview

**Fig. 1:**
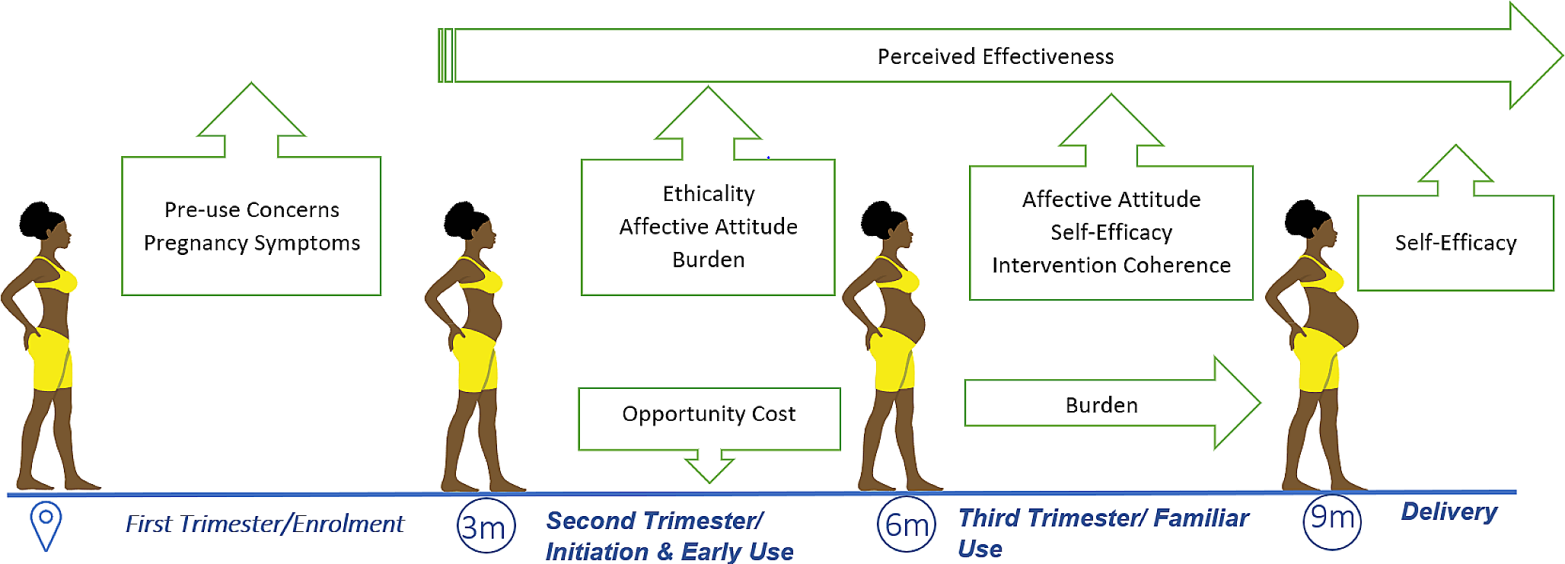
Change in components of product acceptability (per TFA) over three trimesters of pregnancy

Overall, acceptance of study products evolved as pregnancy progressed and participants became more familiar with the products. We organized the results below according to the TFA constructs that emerged within each trimester of pregnancy (definitions in Table 3). We emphasized the commonly mentioned constructs that emerged in each trimester of pregnancy, as depicted in Fig. 1. Upward arrows indicate increase, downward arrows indicate a low/decreased prevalence, and straight arrows signify consistency across trimester(s). For example, for both products, burden increased in the second trimester and persisted in the third, while perceived effectiveness remained consistent during product use.

**Table 3:** The seven domains of the theoretical Framework of Acceptability

Construct	Definition	Example IDI Guide Topics and Discussion Points
Affective Attitude	Assesses an individual’s emotional response to the intervention	Initial feelings about study products (e.g., concerns), current thoughts on the assigned study product
Burden	Focuses on the perceived effort required for participation in the intervention	Challenges with product use, facilitators to using assigned product consistently
Ethicality	Examines how well the intervention aligns with personal values	How product use fits into their and other persons in the social networks (e.g., healthcare providers, sexual partners, family, peers, etc.) beliefs and values
Intervention Coherence	Measures the participant’s comprehension of how the intervention works	Perceived effect of product use on maternal and child health now and in the future
Opportunity Costs	Evaluates what needs to be sacrificed for engagement in the intervention	Impact of product use on own or partner’s sexual desire/pleasure
Perceived Effectiveness	Evaluates the likelihood of the intervention achieving its intended purpose	Satisfaction with study product in preventing HIV during pregnancy, desire to use study product in the future
Self-Efficacy	Pertains to the participant’s confidence in their ability to carry out the required behaviors for intervention participation	Changes in ability and confidence related to ring insertion or pill use during pregnancy, plans and confidence for ring removal before delivery, confidence to remove the ring during pregnancy, experiences with ring removal during delivery

### Pre-use Concerns

Initially, concerns about the ring included potential interference with sexual activity, loss in the vagina, large size and shape, dislodging during sex, falling out during daily activities, weight gain, and discomfort due to improper insertion. Concerns about pill usage included forgetting to take them and difficulties with swallowing. For both products, fears of potential side effects pertained to both the mother and the unborn baby:

*“I was anxious about the ring…I was like, is it [ring] not going to cause some problems on my vagina? Or…some problems on my child or maybe having a stillbirth.” [760-94025-2, Nulliparous, Ring, Blantyre, Early third trimester]*.

*“At first I thought ‘will these pills not going to kill my child?’” [760-81275-8, Parous, Pills, Blantyre, Second trimester]*.

Importantly, while some participants had experienced pregnancy symptoms (e.g., vomiting, nausea, dizziness, and fatigue), they typically had resolved before initiating product use, and thus, these symptoms didn’t taint initial experiences with study products. Additionally, a few participants had no initial concerns with either product, as expressed here: *“I wasn’t worried because I was protecting my life from getting HIV, so I was not worried at all.” [753-43934-8, Nulliparous, Ring, Kampala, Early third trimester].* Several participants found relief in witnessing healthy babies in previous cohorts, while others credited study counselors for addressing initial fears, but ultimately found relief through their own experiences over time:

*“I saw my friends who have healthy babies who also joined this study and the children are growing without facing any challenges.” [760-58411-7, Nulliparous, Ring, Blantyre, Second trimester]*.

*“The study staff said, ‘you are not the first to be in the study, we have enrolled other participants in the previous cohorts. We have not encountered anyone who has experienced a problem of the ring proceeding further into the stomach.’ It helped me to be at peace…” [897-57841-9, Parous, Ring, Johannesburg, Second trimester]*.

### Product Initiation and Early Use

#### Ethicality and Affective Attitudes

Early in the study, ethical concerns frequently arose, primarily related to disclosure to partners and intertwined with participants’ attitudes towards the products. Most ring users disclosed their use and voiced that it should be a collaborative decision-making process or that partners should offer support since it involved the welfare of the child. One participant stated, *“I also needed approval from my partner that I can use the ring because it is not only one sided, because we both had the same thought of how it would affect the baby.” [Nulliparous, Ring, Johannesburg, Early third trimester]* Ring users who didn’t disclose appreciated the ring’s discreet nature, despite initially fearing it could be felt during sex:

*“I thought I would feel it during sex but no I do not feel it at all. He [partner] does not know that I have the ring and he has never told me anything about feeling something there.” [897-96741-6, Nulliparous, Ring, Johannesburg, Early third trimester]*.

Pill users who didn’t disclose typically cited concerns about potential confusion with HIV treatment. Similar to ring users, those who disclosed believed it was a shared responsibility between both partners.

Despite initial hesitancy and concerns, most partners became supportive after learning more about the study, study products, and the baby’s post-birth monitoring: *“I conversed with my husband, but initially he had refused saying that the tablets might affect the baby but when I told him that afterwards they also monitor the baby he eventually accepted.” [753-17251-6, Parous, Pills, Kampala, Second trimester].* While many ring users had positive attitudes of the ring due to discreteness and regimen, a few pill users disliked daily intake and found the pills challenging to swallow due to size.

#### Burden – Early Challenges and Uncertainty

In the early stages of product use, many participants reported side effects. Ring users commonly reported vaginal itchiness and discharge. Less common events included vaginal bleeding, diarrhea, vomiting, smelly urine, abdominal pain, dizziness, and headaches. Pill users mostly reported nausea, headaches, and dizziness. These symptoms typically appeared within the first few weeks of product use and resolved quickly.

*“The first time that after inserting the ring, I think for two days since it was something new in my vagina, I experienced some slight itchiness.” [774-62976-5, Parous, Ring, Chitungwiza, Second trimester]*.

*“The first time they gave me a pill and I took it from here…In the evening, I felt dizzy, nausea. I just knew that after some days, it would get used to the body…These side effects lasted for 3 days before they disappeared.” [753-88180-4, Nulliparous, Pills, Kampala, Second trimester]*.

Some participants were unsure if side effects like vaginal discharge were due to product use or pregnancy itself. Reassurance from staff that these side effects were normal and that other participants had used the study products before, provided comfort. Moreover, a ring user had an issue with her partner feeling the ring during sex, but it was resolved after consulting study staff and pushing the ring deeper. In contrast, a nulliparous participant removed her first ring during sex at the request of her partner because *“he was feeling it during sex and he was afraid of it.” [760-21446-1, Nulliparous, Ring, Blantyre, Second trimester]* She was offered a counseling session for her partner but upon discussion with him, the issue resolved itself.

### Changes in Product Use Experiences and Pregnancy Progression

#### Affective Attitude

With continued use, both ring and pill users reported satisfaction with their assigned products, driven by various reasons. Participants felt a strong duty to protect their unborn children, motivating their study participation. Ring users found the ring highly acceptable as it seamlessly integrated into their daily routines, didn’t change sexual pleasure, offered discreet protection, had a longer duration of use, required minimal effort to use, and provided peace of mind knowing that the ring was always *in-situ*, unlike the daily pills, as conveyed by this ring user:

“*I was satisfied, as long I knew it [ring] was there, then I was like having that feeling that I am protected…like the pills you have to remember to drink it at 9 a.m.… the ring was just a relief that you only insert it once and remove it month end. You forget it [the ring] and continue with your life.” [897-11007-4, Parous, Ring, Johannesburg, Early third trimester]*.

The initial fears about the ring subsided as participants realized their concerns were unfounded. Discreetness was appreciated by those who hadn’t disclosed its use to their male partners, and some liked the increased vaginal wetness they experienced with the ring, which improved their sexual experiences. Similarly, pill users were typically satisfied with daily use, appreciating the flexibility in setting their own time, especially if they were already on other medications. The assurance of their child’s health and well-being, often confirmed by the fetus’ movement, reinforced their acceptance of both the ring and pills.

*“I feel so excited because ever since I started taking it [pill], it did not give me any problems. Or for me to say, ‘This pill caused me this, or to that my baby has stopped playing’.” [774-51372-6, Parous, Pills, Chitungwiza, Second trimester]*.

“*So far it [ring] has not caused me any problems because the baby moves well in the womb, he did not stop moving in the womb when I started using the ring.” [753-51840-4, Nulliparous, Ring, Kampala, Early third trimester]*.

#### Self-Efficacy

Although not the most dominant TFA construct, self-efficacy played a significant role in participants’ adherence to the study products during their pregnancy and reflected their confidence in managing the products themselves. Most ring users were comfortable and capable of self-removal, but when it came to insertion, preferences varied. The majority of ring users preferred learning how to insert the ring themselves. They believed that by doing so, they could have greater control over its removal, especially at the onset of labor pains, in cases where the ring might fall out, or simply due to their discomfort undressing in front of a clinician. Some participants inserted the ring themselves but still sought validation from a study clinician about its placement, highlighting the importance of clinical guidance. On the other hand, a small number of ring users preferred a study clinician to perform the insertion to ensure correct placement.

*“I think inserting it myself is the right thing because you will find that you have labor pains, the doctors and nurses will not be next to you as when they helped you to insert it…So, it is better to train yourself now while there is still time, you see.” [897-96741-6, Nulliparous, Ring, Johannesburg, Early third trimester]*.

Additionally, most pill users reported no challenges with consistent product use as pregnancy and product use time progressed. Only one participant mentioned splitting the pill in half to make it easier to take, but overall, they found it manageable.

*“I didn’t experience any difficulty just like I told you…I swallow it and continue doing my other things…I set an alarm every time I wake up so that I don’t forget to take my pills. The moment the alarm goes off, I look for my medication and take it.” [753-88180-4, Nulliparous, Pills, Kampala, Second trimester].*

#### Burden and Opportunity-cost

The construct of *burden* related to product use was rarely mentioned. Some ring users experienced side effects like vaginal discharge and itchiness throughout their product use journey, but these were generally tolerable. Although many participants reported no side effects or no issues with ring use, some frequently checked the ring during baths, though this habit often diminished after several months. A few participants felt burdened by ring use as their pregnancies progressed due to challenges with bending to insert and remove the ring.

*“I at first found it difficult because you can see my pregnancy is advanced and so bending to touch down [inside the vagina] is difficult. At first I failed, I tried and it refused, but I removed it at last.” [753-57174-1, Nulliparous, Ring, Kampala, Early third trimester]*.

*“When I first put on this ring, it was easy for me but removing it was a bit challenging…I noticed that it was very far because the tummy is now low like this [touching her tummy]… When I removed it, I lied down. I had to lie down looking up, that is when I removed it.” [774-36641-6, Parous, Chitungwiza, Ring, Early third trimester]*.

However, study staff assistance for insertion or removal during pregnancy was infrequent, as most participants managed independently. Some nulliparous participants worried about delivery due to lack of experience, and others were concerned about potential harm to the baby if the ring wasn’t removed during childbirth. One participant planned to have her husband assist with ring removal during labor.

*“What I think about it is how to remove it when labor pains start because my finger is short and cannot reach the ring. The finger failed to reach where the ring is and I was told that when the labor pains begin, I have to remove the ring…I plan to tell my husband to remove it.” [753-51840-4, Nulliparous, Ring, Kampala, Early third trimester]*.

Furthermore, beyond the delivery process itself, some participants worried that the ring could potentially affect their children’s health in the future, despite observing no side effects during several months of use while pregnant. However, of the 4 ring users who delivered before the interview, three parous participants found the ring removal process easy. While one removed the ring the day before delivery, the other two removed it at the onset of labor. One of these participants believed the ring initially hid the labor signs, causing her to be unaware of her condition until she removed it: *At first it [ring] hid the sign. I was feeling pain in the stomach and I was wondering why. My heart told me to go to the toilet and check so when I went there I removed it [ring] and all the signs for delivery started.” [753-70400-1, Parous, Ring, Kampala, Second trimester]* In contrast, a nulliparous participant expressed confidence in her ring insertion and removal but found *“it painful to remove”* during labor. Among pill users, a few experienced headaches or dizziness throughout their product use and one user worried that because the pills were not localized and “*circulates in the blood*” she “*could not rule out some problems*” on her unborn child. *[760-79852-5, Parous, Pill, Blantyre, Early third trimester]* Lastly, while all parous and most nulliparous participants preferred their assigned study product, a few nulliparous pill users said they would choose the ring in the future, either to try it out or because they disliked taking pills daily. Only one nulliparous ring user, was reluctant to use the ring in the future due to experienced side effects.

Participants seldom discussed opportunity cost, but when they did, it typically revolved around sexual activity or disclosure. Participants rarely mentioned male partners feeling the ring during sex, and when it did come up, it was often resolved by re-adjusting the ring. However, there were some challenges related to sex. Two participants mentioned partners refusing to have sex due to the ring, while another sacrificed her usual sexual pleasure to wear it because she experienced pain while having sex with the ring inserted. Additionally, one partner requested a participant remove the ring before every sexual encounter, which she complied with despite being counseled by study staff to keep the ring in place.

#### Perceived Effectiveness and Intervention Coherence

Participants typically indicated they regarded the study products as providing near-universal protection to themselves and their unborn children. However, some issues regarding intervention coherence emerged. Most ring users recognized that the ring wasn’t 100% effective and understood the need to use it as prescribed, as described here: *“if you remove it [ring]”* then *“the drug might fail to work.” [ 774-59740-7, Nulliparous, Chitungwiza Ring, Second trimester]* However, some lacked a clear understanding that the ring was a drug-dispensing device and believed if the ring wasn’t specifically placed in a particular location in the vagina it would lose its effectiveness. Concerns were also voiced among some ring users that the ring could get stuck on the baby’s head and cause issues during delivery if not removed before labor, as voiced here: *“I may become weak and fail to remove it. If I fail to remove it quickly, the baby may fail to come out because of the ring.” [753-26814-6, Parous, Ring, Kampala, Early third trimester]* Furthermore, there were also topics pertaining to intervention coherence that emerged only among parous participants. For example, a few parous participants had a belief that the ring’s vaginal localization wouldn’t harm the baby, because there was no drug exposure to the baby. One participant shared, *“Like where the baby is at is closed. So, nothing reaches my baby, it [the ring] is inside of me… The ring doesn’t reach my baby because my baby is protected there in the uterus as there is water and its closed.” [897-36555-3, Parous, Ring, Johannesburg, Second trimester]* In contrast, a couple of participants also shared misperceptions that the drug released from the ring would continue to provide protection up to a year after product discontinuation, or that the ring only protected the baby and not the user – likely due to the staff’s advice to use condoms alongside the study products, as voiced here: “*No. It does not protect her [the user] because we are taught that we have to protect ourselves. That the product they give us protects the baby and not the adult person*.” *[753-92922-5, Parous, Ring, Kampala, Early third trimester]* Lastly, a participant removed the ring for cleaning despite staff advice not to do so. She stated, “*I would say to myself, “let me clean the ring because I cannot go for a month without cleaning it… Firstly, I start by removing the ring, then I wash it. Then I clean my vagina and re- insert the ring.” [774-62976-5, Parous, Ring, Chitungwiza, Second trimester]* Among pill users, all understood the mechanism of action of oral PrEP.

#### Ethicality

Ethicality concerns remained as participants looked towards the future, beyond study participation and pregnancy. Some participants stressed that their responsibility to protect their child from HIV extended beyond childbirth, which emphasized that delivery didn’t mark the end of this duty. Participants wished for the study products to be available during lactation too, as they believed they were still at risk for HIV acquisition and expressed concerns about the safety of their breastfeeding infants.

*“I am worried that I may get HIV during breastfeeding because I won’t be having the ring and the baby may get HIV. I cannot be sure of what my husband does when he is away from home.” [753-51840-4, Nulliparous, Ring, Kampala, Early third trimester]*.

*“We were now used. And that when your baby will be breastfeeding, they will be breastfeeding on the milk that is safe. But now if you stop using the pills, you wouldn’t know in the future whether you are giving your baby contaminated milk [Milk with HIV infection] or not, or whether the milk is still safe or not.” [774-51372-6, Parous, Pills, Chitungwiza, Second trimester]*.

Though rare, a few participants did oppose the use of study products post-pregnancy as they believed it unnecessary since the baby would be born-HIV negative and thus had low risk of HIV acquisition. Furthermore, participants still held concerns about convincing their partners of their continued use, beyond pregnancy. They felt that partner support for ongoing product use was often motivated by the desire to protect the baby, and they anticipated challenges in maintaining partner trust and fidelity if they continued to use HIV prevention when not pregnant. Overall, no major differences by site or country were found in this analysis regarding product acceptability.

## Discussion

DELIVER participants in the late third trimester found the ring to be generally acceptable when used briefly during pregnancy [20]. This analysis extends those results by examining multi-month use of the ring and oral PrEP, in the second and third trimesters of pregnancy. We observed several common themes among all participants across the pregnancy stages. Participants showed overwhelming positive attitudes toward the study products, considering them user-friendly and minimally burdensome. Participants were satisfied with their assigned products, which seamlessly fit into their daily lives, and ring users appreciated its monthly regimen and discreetness. All participants reported peace of mind because they understood consistent use ensured their protection against HIV. They further expressed it was their maternal duty to protect the unborn child against HIV which facilitated persistent use. While participants held some concerns about product use, similar to findings among non-pregnant persons [10, 25–27], these concerns decreased overtime. Most participants disclosed to male partners as they believed the well-being of the fetus was a shared responsibility, and ultimately most partners turned out to be supportive. Moreover, a majority of participants were comfortable inserting and removing the ring, despite pregnancy progression, with only a few challenges being reported. Finally, there were a few occurrences of misunderstandings about the ring’s mechanism of action, but none were noted for oral PrEP, a more familiar prevention strategy.

There were some notable differences among participants in the second trimester and early third trimester compared to the late third trimester. First, there was evidence of experiential learning, where participants reported a reduction in their fears after hearing the successful experiences of participants and their babies from previous cohorts. Perceived effectiveness was strong among participants in this analysis, with no doubts expressed about either study products’ ability to protect against HIV. This differed from the late third trimester, where some participants awaited post-delivery HIV-negative test confirmation before making such a determination. Lastly, while a comparison of labor/delivery experiences was not examined in late third trimester, we noted slight parity-related differences in anticipated delivery experiences, primarily stemming from a lack of understanding about labor/delivery among several nulliparous participants. Actual labor experiences varied among the few that delivered before partaking in the IDIs; a nulliparous participant reported pain during ring removal while in contrast three parous participants found the ring removal easy during labor. A few parous participants had a distinct interpretation of the ring’s effects on the baby, believing that the ring’s localization ensured the fetus safety since it wasn’t in direct contact with the ring. Overall, no major differences by site or country were found in these perceptions and concerns regarding the acceptability of the ring or oral PrEP. While this suggests a broad consensus on the acceptability of these HIV prevention methods across different locations, there is a need to confirm these findings in individual country settings and in areas within the study countries where the research didn’t take place, such as other provinces and/or rural areas.

The study’s findings underscore the complex interplay between ethical considerations, disclosure to partners, and participants’ attitudes towards product use during pregnancy. Similar to prior studies, participants felt a strong maternal duty to protect their unborn children’s health, motivating them to use PrEP during pregnancy [28–30] despite concerns remaining about the benefits and risks of using the study products while pregnant [30, 31]. The novelty of the ring raised more worries, although observing or hearing about successful usage by other pregnant persons, resulting in healthy births, helped ease these concerns. Concerns parallel the MTN-043 study where lactating persons initially favored the pills due to familiarity but later voiced preference for the ring, after gaining product experience [25]. This hesitancy with new products during pregnancy resembled the cautious approach to the COVID-19 vaccine, where pregnant and lactating persons were hesitant to get vaccinated due to limited safety evidence and fear of harm to the fetus or infant [32, 33]. As the ring comes to market, “ring ambassadors” or peer educators can be instrumental by sharing positive stories, offering support, and conveying personal experiences to dispel patient’s fears and misconceptions about product use during pregnancy [34]. Moreover, ongoing surveillance of ring use during the perinatal period is essential for a robust evidence base and addressing emerging safety concerns that can be accurately disseminated via trusted communication channels. However, experiential learning and direct product experience did not eliminate all fears, particularly regarding the child’s future well-being. Effectively addressing and mitigating these concerns, possibly through periodic check-ins and education, are critical for intervention success during and after pregnancy. Lastly, continuous post-partum support from consistent healthcare providers is essential for mothers and infants, ensuring a smooth transition and addressing ongoing needs in the crucial period following childbirth.

The ethical dimension of disclosing product use to male partners varied among participants, with some stressing its importance for the child’s health and others fearing prohibitions or discouragement. Partner disclosure, a common barrier to oral or vaginal PrEP use in other studies [31, 35, 36], often prompts hesitancy and concerns, primarily related to potential dyadic conflicts [31, 36]. However, as previously found, most male partners became supportive after understanding the importance of safeguarding the child’s health [31, 36], with additional information and staff support helping to alleviate their fears. Acknowledging varied individual preferences and couple dynamics, interventions should employ multifaceted strategies that involve male partner participation when appropriate and address concerns to balance maternal autonomy and shared responsibility. This underscores the ethical complexities pregnant persons face when using these products and the crucial role healthcare providers play in facilitating informed decisions. High discontinuation rates of prevention methods among people of reproductive age, a significant barrier to HIV risk reduction, can be mitigated by male partners who can offer ongoing encouragement and reminders, motivating this population to continue product use despite side effects [36]. Supporting discreet product use also highlights ongoing efforts to combat HIV-related stigma and equip birthing persons with strategies to prevent intimate partner violence when they choose not to disclose, as anticipated stigma and limited disclosure have been linked to PrEP discontinuation and adherence challenges [37].

Moreover, some participants talked about side effects they experienced with product use and with some worries that these side effects could reoccur if they used the products after delivery. Timely support from healthcare providers is essential to address side effects and ease anxieties. Strategies should be established to promptly address concerns regarding side effects on both the mother and unborn child; however, we found that product-related side effects during pregnancy resolved over time with continued product use as seen in non-pregnant persons [38, 39]. Challenges with ring insertion and removal during advanced pregnancy was noted for some, emphasizing the importance of healthcare providers offering timely assistance and support. Proper training and education of healthcare providers is crucial to ensure they have adequate knowledge to assist with ring insertions and removal, when needed [40].

Lastly, the study shows birthing persons strong interest in using the ring during pregnancy, underscoring the crucial importance of offering them a variety of HIV prevention options. Emerging long-acting PrEP options will further enhance the range of methods available, allowing them to choose what best fits their lifestyles and needs. Our findings provide valuable insights for the scale-up and introduction of the DPV ring and other newer agents and delivery forms into the general population. The strong acceptability and positive attitudes toward the ring and oral PrEP among pregnant persons in this study indicate that similar PrEP methods could be well-received. Evidence of experiential learning and the reduction in fears after hearing about successful usage suggest that positive testimonials and real-world evidence will be critical in promoting both the ring and other long-acting agents in pregnant persons. The scale-up of the ring and other PrEP methods could benefit from leveraging peer educators and ‘product ambassadors’ from various populations, such as perinatal and non-perinatal individuals [34], to share experiences and provide support, helping to mitigate initial fears and misconceptions. Furthermore, it is crucial for policy makers to recognize the high acceptability of the ring among this population and ensure it is incorporated into their agendas to increase the accessibility of prevention options for this population.

Several limitations should be considered in interpreting this study’s findings. First, data collection occurred during the COVID-19 pandemic, with participants and interviewers following safety measures, including the use of facemasks, potentially affecting the quality of interviews. Second, interviews occurred in advanced stages of the participants’ pregnancies, possibly impacting their responses due to physical discomfort or fatigue. Third, social desirability bias could have influenced responses as participants were users of the studied products. Fourth, the study’s generalizability is limited as it was a trial with self-selected participants from specific recruitment channels. Fifth, the study was conducted in well-established urban clinic settings; therefore, there is a need to conduct research in clinic settings in rural areas and/other provinces in these countries. However, despite these limitations, the study findings enhance our understanding of the acceptability of the ring and pills during pregnancy and offer insights into product challenges and opportunities. This contributes to the knowledge of maternal HIV prevention and has implications for developing interventions to promote maternal and child health.

## Conclusions

This qualitative study revealed that with a strong maternal responsibility driving consistent use, pregnant persons found the ring and oral PrEP easy to use without significant sacrifices to their daily lives. Study staff played a vital role in addressing participants’ concerns about the study products, although direct and indirect experiences with the products over time proved to be key factors in alleviating these concerns too. These novel insights into the challenges and opportunities of PrEP use during pregnancy emphasize the need for comprehensive client-centered counseling, support, and education for pregnant persons considering PrEP. Overall, this research offers promising prospects in a population that greatly requires such prevention options, contributing to our understanding of maternal HIV prevention.

## Data Availability

Data are available through the Microbicide Trials Network.

## Declarations

### Study Products

The dapivirine vaginal rings used in this study were developed and supplied by the International Partnership for Microbicides (IPM). Oral tenofovir disoproxil fumarate/emtricitabine (TDF/FTC) was donated by Gilead Sciences.
